# Deviations from Expectations: A Commentary on Aliev et al.

**DOI:** 10.1007/s10519-018-9891-5

**Published:** 2018-02-21

**Authors:** Sophie van der Sluis, César-Reyer Vroom, Conor V. Dolan

**Affiliations:** 1 Department of Clinical Genetics, Section Complex Trait Genetics, Center for Neurogenomics and Cognitive Research, Amsterdam Neuroscience, VU Medical Centre Amsterdam (VUmc), Amsterdam, The Netherlands; 2 Department of Complex Trait Genetics, Center for Neurogenomics and Cognitive Research, Amsterdam Neuroscience, VU University Amsterdam, De Boelelaan 1085, 1081 HV Amsterdam, The Netherlands; 3 Department of Biological Psychology, VU University Amsterdam, Amsterdam, The Netherlands

The Trait-based Association Test that uses Extended Simes (TATES, Van der Sluis et al. 2013) was proposed as a multivariate test in the context of genome-wide association studies (GWAS). In regular univariate GWAS, the statistical association between a phenotype of interest (e.g., height) and a single nucleotide polymorphism (SNP) is tested, yielding a single p-value. If *m* phenotypes are studied, each phenotype can be individually regressed on the SNP, yielding *m* p-values. TATES is a so-called combination test: it tests a multivariate hypothesis by combining the *m* p-values obtained in the *m* univariate tests. Specifically, TATES is based on selection of the minimal p-value among *m* appropriately weighted p-values, and as such tests the hypothesis that at least one of the *m* phenotypes is associated to the particular SNP. TATES was inspired by the GATES procedure, a gene based test of association (Li et al. 2011).

Aliev et al. set out to demonstrate that the Type I error rate of TATES is incorrect. To this end, they present results of a small simulation study in which they examined the empirical Type I error rate of TATES given two or three correlated phenotypes, and a mathematical proof showing that the distribution of TATES p-values is not uniform under the null-hypothesis (H0) given two phenotypes.

We gratefully use this opportunity to comment on this work.

## Empirical Type I error rates

In the original TATES paper, the authors showed in 20 scenarios (8 of which concerned the effect of missing data) that the Type I error rate of TATES is correct when the number of phenotypes *m* equaled 20, the number of simulations *Nsim* equaled 2000, and *α* was set to 0.05. These simulation settings were deemed realistic in the context of questionnaire data (i.e., psychological questionnaires often consist of > 10 items, which one may want to study individually), and tailored to this context, featuring various realistic models to account for the phenotypic covariance structure (specifically, 1-, 2- or 4-factor models and network models).

Aliev et al. report simulations featuring *m* = 2 and *m* = 3 phenotypes in 10 correlational settings. In these simulations, 6 out of 10 (*m* = 2), and 21 out of 30 (*m* = 3) phenotypic correlations *r* > 0.70 (see Aliev et al., Table 1, column 4, and Table 2, column 5). They then show that, given *Nsim* = 100,000, the Type I error rate of TATES deviated significantly from 0.05 (95% confidence interval: CI_95_ = (0.04865, 0.05135)) in 8 of the 10 scenarios when *m* = 2, with a maximal empirical rate of 0.0553 (when *r* = 0.9343) instead of expected 0.05. When *m* = 3, the Type I error rate of TATES deviated significantly from 0.05 in all 10 presented scenarios, with a maximal empirical rate of 0.0540 (when *r*_1,2_ = 0.81, *r*_1,3_ = 0.95, *r*_2,3_ = 0.78). Before we dwell on the statistical and practical significance of deviations of this magnitude, we first wish to gain a more comprehensive view of TATES’s Type I error rate.

**Table 1:** Type I error rates of four combination tests in 20 simulation scenarios

Correlations	TATES	Simes	minP_Bonf_	minP_NS_
Nvar = 2				
0.1	0.05012	0.05006	0.04932	0.04955
0.3	0.05009	0.04887	*0.04804*	0.05033
0.5	*0.05230*	*0.04858*	*0.04704*	*0.05326*
0.7	*0.05172*	***0.04408***	***0.04153***	***0.05459***
0.9	***0.05550***	***0.04191***	***0.03672***	***0.05967***
Nvar = 4				
0.1	0.05000	0.04987	0.04890	0.04925
0.3	*0.05148*	0.04976	*0.04807*	0.05123
0.5	0.05128	*0.04579*	***0.04291***	*0.05268*
0.7	0.05115	***0.04089***	***0.03637***	***0.05583***
0.9	*0.05324*	***0.03511***	***0.02654***	***0.06250***
Nvar = 8				
0.1	0.05025	0.04999	0.04883	0.04930
0.3	0.05005	*0.04777*	*0.04579*	0.04955
0.5	0.04924	***0.04350***	***0.03968***	0.04949
0.7	*0.04742*	***0.03699***	***0.03135***	*0.05175*
0.9	*0.04495*	***0.02881***	***0.01821***	***0.05524***
Nvar = 16				
0.1	0.04977	0.04945	*0.04803*	*0.04851*
0.3	0.04891	*0.04659*	***0.04363***	*0.04773*
0.5	*0.04674*	***0.04110***	***0.03651***	*0.04664*
0.7	***0.04093***	***0.03209***	***0.02576***	***0.04408***
0.9	***0.03677***	***0.02502***	***0.01314***	*0.04660*
Mean (SD)	0.0491 (0.0042)	0.0428 (0.0076)	0.0388 (0.0108)	0.0514 (0.0045)
Largest overshoot	0.0055	0	0	0.0125
Largest undershoot	0.0132	0.0250	0.0369	0.0059
Sum of absolute deviations across all conditions	0.0525	0.1439	0.2236	0.0664

Italicized values lie outside the 95% confidence interval given Nsim = 100,000 (CI_95_ = 0.0486–0.0514). Italicized and bold values lie outside the 95% confidence interval given Nsim = 10,000 (CI_95_ = 0.0457–0.0543).

### Comprehensive simulations

To investigate the empirical Type I error rate of TATES more extensively, we ran additional simulations in which we varied both the number of phenotypes *m* and the correlations *r* between the *m* phenotypes. Specifically, we simulated data for *m* = 2, 4, 8, and 16, and *r* = 0.1, 0.3, 0.5, 0.7, and 0.9, resulting in 20 simulation settings in total. Note that the resulting correlational structure is compound symmetric (i.e., all phenotypes correlated equally strong), which is consistent with a single (parallel) factor model. We simulated phenotypic and genotypic data for *N* = 2000 subjects. Like Aliev et al., we simulated a single diallelic variant (unassociated, *MAF* = 0.5), and ran *Nsim* = 100,000 simulations for each setting.

To broaden the present scope of our simulations and to put the TATES results into perspective, we analyzed the simulated data using TATES and three other combination tests that, like TATES, are based on selection of the minimal p-value among *m* weighted p-values.

The first combination test that we consider is based on Bonferroni correction (referred to as minP_Bonf_; Simes 1986). Running *m* univariate analyses to regress *m* phenotypes on a SNP, the *m* p-values are all Bonferroni-corrected (i.e., weighted with *m*), and then the smallest Bonferroni-corrected p-value is selected. The second combination test, which we refer to as minP_NS_, is similar to minP_Bonf_, except that one does not correct for the observed number of phenotypes *m*, but for the effective number of phenotypes *M*_eff_. As suggested by Nyholt (2004, based on Šidák 1968, 1971), we calculated *M*_eff_ based on eigenvalue decomposition of the *m* x *m* phenotypic correlation matrix, and the smallest weighted p-value is selected as minP_NS_ p-value. Note that, assuming non-zero phenotypic correlations, *M*_eff_ is always < *m*, and minP_NS_ is thus always less strict than minP_Bonf_.

The third combination test is the original Simes test that TATES is a variation on (Simes 1986). In Simes, the *m* p-values are first sorted ascendingly. In an iterative fashion, each *j*th p-value of the *m* sorted p-values is then weighted with *m*/*j*, such that the lowest p-value is weighted with the largest weight (i.e., *m*/1) and the highest p-value is weighted with the smallest weight (i.e., *m*/*m* = 1). The Simes p-value then corresponds to the smallest weighted p-value.

TATES, which is based on GATES (Li et al. 2011), weights in fashion similar to Simes, except that the observed number of p-values *m* and *j* are replaced with the effective number of p-values *m*_e_ and *m*_ej_. Specifically, TATES weights each *j*th p-value *p*_j_ by *m*_e_/*m*_ej_, and *m*_ej_ is calculated as

$${m_{ej}}=j - \mathop \sum \limits_{{i=1}}^{j} I({\lambda _i}>1)({\lambda _i} - 1)$$

where *j* is the number of top *j* p-values, λ_i_ denotes the ith eigenvalue of the correlation matrix between the *j* p-values (which can be approximated from the correlations between the *j* phenotypes), and I(x) is an indicator function taking on value 0 if λ_i_ ≤ 1 and 1 if λ_i_ > 1. That is, the effective number of p-values *m*_ej_ among the *j* p-values is calculated as the observed number of p-values *j* minus the sum of the difference between the eigenvalues λ_i_ and 1 for those eigenvalues λi > 1. The value of *m*_e_ is equal to *m*_ej_ for the case that *j* = *m*, i.e., when the selection of top p-values covers all p-values. The TATES p-value then corresponds to the smallest weighted p-value. Following this procedure, the smallest original p-value is always weighted by the largest weight, while the largest original p-value is weighted by *m*_e_/*m*_e_ = 1 as in that case *m*_ej_ = *m*_e_. As the weight *m*_e_/*m*_ej_ is always ≥ 1, the weighted p-values are, like in the Simes test, always ≥ the original, unweighted p-values.

All four combination tests test the hypothesis that at least 1 of the *m* phenotypes is associated to the SNP under study by assessing whether the selected weighted p-value is smaller than a beforehand established threshold (it being 0.05, or the default genome-wide threshold 5 × 10^−8^).

We ran the 20 simulation scenarios for all four methods, and then established the percentage of p-values per scenario smaller than 0.05. For all four methods, these observed Type I error rates are shown in Table 1. We then established whether the observed percentage fell inside the CI_95_ given *α* = 0.05. The standard error of the ML estimator of the p-value is $SE=\sqrt {p \times (1 - p)/Nsim}$, where *p* denotes the percentage of significant tests expected given the chosen *α* (i.e., 0.05), and *Nsim* the total number of simulations. Given *Nsim* = 100,000 and *α* = 0.05, the CI_95_ thus equals (*p* − 1.96 × SE, *p* + 1.96 × SE) = (0.04865, 0.05135). In Table 1, values that fall outside the CI_95_ given Nsim = 100,000 are italicized, while values falling outside the CI_95_ given *Nsim* = 10,000 are italicized and bold.

The Type I error results in Table 1 show that when correlations are medium-to-high, TATES is slightly liberal when *m* is small, yet slightly conservative when *m* is large. Simes and minP_Bonf_ are almost always conservative, and becomes more so with increasing correlations and increasing *m*. In contrast, minP_NS_ is generally liberal, and especially so when *m* is small and correlations are high. If we sum the absolute deviations from 0.05 across all 20 scenarios, we see that overall, TATES remains closest to 0.05, while minP_Bonf_ shows the largest overall deviation, entirely due to its conservativeness. Indeed, minP_Bonf_ shows the largest undershoot, while minP_NS_ shows the largest overshoot.

Overall, the Type I error rate of TATES does show *m-* and *r*-dependent variation around 0.05, but these deviations are small, especially compared to the other considered combination tests. The deviations reported by Aliev et al. are associated with the special case of small *m* and (very) high *r*. However, regardless of the narrow scope of the simulations by Aliev et al., one may still ask whether the observed deviations are a reason to reject the TATES procedure.

### Power to detect departures from nominal α

The larger the number of replications in a simulation study, the more power one has to demonstrate that the empirical Type I error rate of a method deviates from the expected Type I error rate. For instance, with *Nsim* = 2000 (original TATES paper), *Nsim* = 10,000, and *Nsim* = 100,000 (Aliev et al., and present simulations), the CI_95_’s of an unbiased p-value are 0.0404–0.0596, 0.0457–0.0543, and 0.0486–0.0514, respectively. The empirical Type I error rates of TATES, as displayed in Table 1, should thus be considered incorrect in either 1, 3, or, 10 of the 20 scenarios, depending on the power, i.e., the chosen *Nsim*. Aliev et al. emphasized statistical significance in assessing the Type I error results. However, in this situation, we believe that it is more important to consider the practical relevance of deviations of 0.05, 0.005, or 0.001. We are convinced that deviations of this magnitude, while statistically significant given large *Nsim*, cannot justify the rejection of TATES, or any other method, and believe therefore that the TATES Type I error rates give little reason for concern.

## The assumption of uniformly distributed p-values

Aliev et al. argued that the distribution of the TATES p-values should be uniform under the H0, and that the probability distribution function should at least not exceed 1 at the low end of the distribution, i.e., around 0. They consider this a condition for the Type I error rate of TATES to be correct. It is important to note that p-values may not be uniformly distributed under the H0 in special cases (see Aliev et al.’s references to Murdoch et al. 2008; Bland 2013). However, in most statistical tests, when distributional assumptions and sample size requirements are met, the resulting p-values are indeed assumed to be uniformly distributed under H0. Combination tests like minP_Bonf_, minP_NS_, and TATES work with these p-values. However, these combination tests themselves are based on *selection* of the minimal p-value among *m* weighted p-values. So while the p-values of the *m* univariate tests, on which these combination tests are based, should indeed generally be uniformly distributed, the relevant question here is whether the p-values resulting from combination tests’ weighted-selection procedure should be uniformly distributed as well.

Before we discuss the distributions of the p-values of these four combination tests for the 20 simulation scenarios described above, we wish to emphasize the nature of the weighting in the four different tests. In minP_Bonf_ and minP_NS_, all *m* p-values are weighted by the same constant, being *m* for minP_Bonf_ and *M*_eff_ for minP_NS_. In the original Simes test and TATES, however, each of the *m* p-values is weighted differently, i.e., each *j*th p-value among the *m* ascendingly sorted p-values is weighted with *m*/*j* or *m*_e_/*m*_ej_, respectively.

**Fig. 1:**
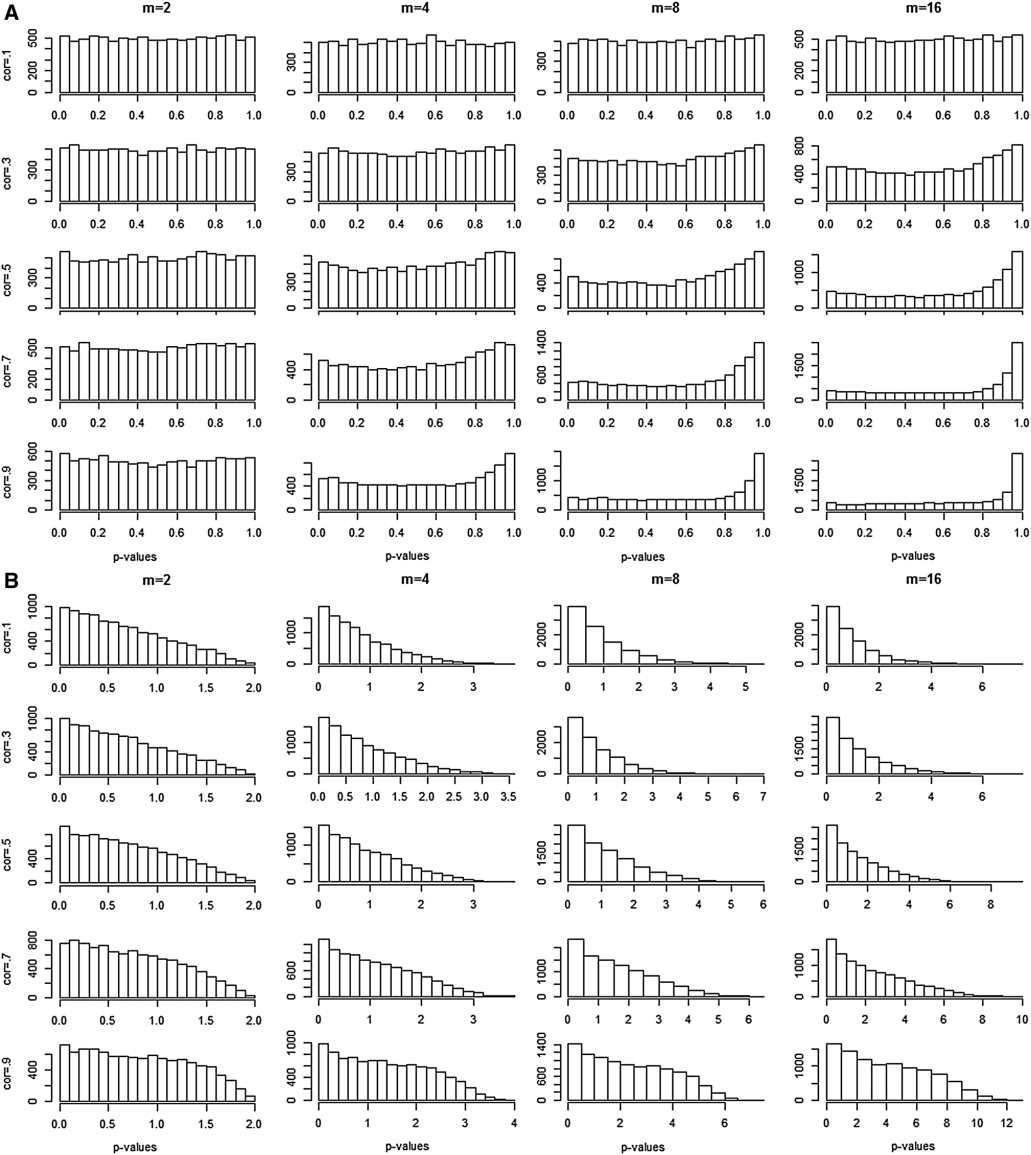
P-value distributions under the H0 for TATES (**a**) and minP_Bonf_ (**b**) in 20 simulated scenarios varying the number of phenotypes *m* (columns) and the correlations between these phenotypes (rows). Note that the p-value distributions of Simes and minP_NS_ are quite similar to those of TATES and minP_Bonf_, respectively, and are therefore not displayed separately

Figure 1 shows the distributions of the first 10,000 (of 100,000) p-values obtained with TATES and minP_Bonf_ (panel a and b, respectively) in each of the 20 aforementioned simulation settings (the p-value distributions obtained with Simes and minP_NS_ are very similar to those of TATES and minP_Bonf_, respectively, and therefore not shown separately).

Clearly, p-value distributions of methods that are based on selection of the minimal weighted p-value are not uniformly distributed under H0, even if the *m* p-values that they are based on are. Specifically, when all p-values are weighted with the same weight (minP_Bonf_, minP_NS_), selection of the minimal weighted p-value results (as could be expected) in a p-value distribution with a right, positive skew. When smaller p-values are weighted more heavily, the deviation of uniformity increases with increasing *m* and increasing correlations, with the bulk of p-values at the high end of the distribution. Indeed, as both Simes and TATES weight the highest original p-value with the smallest weight (i.e., 1, see above), the Simes p-value and the TATES p-value very often equal the largest p-value before weighting.

## Conclusion

Aliev et al. set out to show that the Type I error rate of TATES procedure is incorrect by presenting results of a simulation study and a mathematical proof concerning the non-uniformity of TATES’s p-value distribution. With respect to the former, we believe that the simulation results of Aliev et al. as well as our own showed that TATES’s Type I error rate indeed shows slight in- *or* deflation, depending on the number of phenotypes *m* and the strength of their intercorrelations *r*. However, we consider the observed deviations of 0.05, whether or not statistically significant, to be too small to be considered of practical concern. Note that in this commentary, we addressed the Type I error rate given an expected rate of α = 0.05, like Aliev et al. did. We also considered α = 0.01 and α = 0.001 (Supplemental Table 1) and found that Type I error rates of TATES were close to expectation (ranges 0.0081–0.0120, and 0.00094–0.00129, respectively). While again some values deviated statistically significantly from expectation (specifically, given α = 0.01 and α = 0.001, 1 and 0 values of the 20 simulated settings fell outside the CI_95_ given *Nsim* = 10,000, and 9 and 5 fell outside the CI_95_ given *Nsim* = 100,000, respectively), the deviations were small and, in our view, of little practical significance.

With respect to mathematical proof concerning the uniformity of the p-value distribution, we believe that the assumption that the distribution of p-values of a method that is based on p-value selection should be uniform, is based on misconception. The p-values that the combination tests are based on should be uniformly distributed, but the p-values of subsequent weighted-selection-based combination tests are not expected to be uniformly distributed.

All in all, if one wishes to apply a combination test to tackle a multivariate problem, we believe that TATES represents a viable option.

## Electronic supplementary material

Below is the link to the electronic supplementary material.

Supplementary material 1 (DOCX 19 KB)
