# Supplementary material for: Deviations from Expectations: A Commentary on Aliev et al
Source: Behav Genet. 2018 Feb 21;48(2):168–72. doi: 10.1007/s10519-018-9891-5 (PMC5846829; doi:10.1007/s10519-018-9891-5)
Supplement: Supplementary file 1 — Supplementary material 1 (DOCX 19 KB) [file 10519_2018_9891_MOESM1_ESM.docx]

SUPPLEMENT TO

Deviations from expectations:

A commentary on Aliev et al.

Sophie van der Sluis^1,2*^, César-Reyer Vroom^1,2^, Conor V. Dolan^3^

^1^ Department of Clinical Genetics, Section Complex Trait Genetics, Center for Neurogenomics and Cognitive Research, Amsterdam Neuroscience, VU Medical Centre Amsterdam (VUmc), The Netherlands

^2^ Department of Complex Trait Genetics, Center for Neurogenomics and Cognitive Research, Amsterdam Neuroscience, VU University Amsterdam, The Netherlands.

^3^ Department of Biological Psychology, VU University Amsterdam, Amsterdam, The Netherlands

*Correspondence to:

Sophie van der Sluis: Department of Complex Trait Genetics, VU University, De Boelelaan 1085, 1081 HV, Amsterdam, The Netherlands. Phone: +31 20 598 6833, s.vander.sluis@vu.nl

| Supplemental Table 1.  Type I error rates of TATES in 20 simulation scenarios given α=.05, α=.01, and α=.001 | | | | |
| --- | --- | --- | --- | --- |
|  |  |  |  |  |
|  | **correlations** | **α=.05** | **α=.01** | **α=.001** |
| Nvar=2 | .1 | 0.05012 | 0.01037 | 0.00114 |
|  | .3 | 0.05009 | 0.01032 | 0.00100 |
|  | .5 | *0.05230* | *0.01082* | 0.00114 |
|  | .7 | *0.05172* | *0.01080* | 0.00117 |
|  | .9 | ***0.05550*** | *0.01178* | *0.00129* |
| Nvar=4 | .1 | 0.05000 | 0.00987 | 0.00094 |
|  | .3 | *0.05148* | 0.01042 | 0.00096 |
|  | .5 | 0.05128 | *0.01094* | 0.00115 |
|  | .7 | 0.05115 | *0.01113* | *0.00123* |
|  | .9 | *0.05324* | ***0.01199*** | *0.00124* |
| Nvar=8 | .1 | 0.05025 | 0.01051 | 0.00114 |
|  | .3 | 0.05005 | *0.01068* | 0.00108 |
|  | .5 | 0.04924 | 0.01020 | 0.00113 |
|  | .7 | *0.04742* | *0.01105* | *0.00125* |
|  | .9 | *0.04495* | 0.00974 | *0.00125* |
| Nvar=16 | .1 | 0.04977 | 0.01032 | 0.00106 |
|  | .3 | 0.04891 | 0.00997 | 0.00102 |
|  | .5 | *0.04674* | 0.01001 | 0.00110 |
|  | .7 | ***0.04093*** | 0.00957 | 0.00104 |
|  | .9 | ***0.03677*** | *0.00813* | 0.00101 |
|  |  |  |  |  |
| Mean (SD) | | .0491 (.0042) | .01043 (.00083) | .00112 (.00010) |
| Largest overshoot | | .0055 | .00199 | .00029 |
| Largest undershoot | | .0132 | .00187 | 0 |
| Sum of absolute deviations  across all conditions | | .0525 | .01406 | .00254 |
|  |  |  |  |  |
| Note. Italicized values lie outside the 95% confidence interval given Nsim=100,000 (CI_95_=.0486-.0514). Italicized and bold values lie outside the 95% confidence interval given Nsim=10,000 (CI_95_=.0457-.0543). | | | | |
